# Hydropriming and Osmotic Priming Induce Resistance against *Aspergillus niger* in Wheat (*Triticum aestivum* L.) by Activating *β-1, 3-glucanase, Chitinase,* and *Thaumatin-like Protein* Genes

**DOI:** 10.3390/life12122061

**Published:** 2022-12-08

**Authors:** Summia Gul, Amjad Hussain, Qurban Ali, Intikhab Alam, Rana M. Alshegaihi, Qinglin Meng, Wajid Zaman, Hakim Manghwar, Muhammad Farooq Hussain Munis

**Affiliations:** 1 Department of Biology, Heinrich Heine University, 40225 Düsseldorf, Germany; 2 National Key Laboratory of Crop Genetic Improvement, College of Plant Science and Technology, Huazhong Agricultural University, Wuhan 430070, China; 3 Key Laboratory of Monitoring and Management of Crop Diseases and Pest Insects, Department of Plant Pathology, College of Plant Protection, Nanjing Agricultural University, Ministry of Education, Nanjing 210095, China; 4 College of Life Science, South China Agricultural University, Guangzhou 510642, China; 5 Department of Biology, College of Science, University of Jeddah, Jeddah 21493, Saudi Arabia; 6 Department of Biology and Food Engineering, Bozhou University, Bozhou 236800, China; 7 Department of Life Sciences, Yeungnam University, Gyeongsan 38541, Republic of Korea; 8 Lushan Botanical Garden, Chinese Academy of Sciences, Jiujiang 332900, China; 9 Department of Plant Sciences, Faculty of Biological Sciences, Quaid-i-Azam University, Islamabad 45320, Pakistan

**Keywords:** wheat, priming, *Aspergillus niger*, qRT-PCR, wilting, *TLP*, *chitinase*, *β-1,3-glucanase*

## Abstract

Priming is used as a method to improve plant growth and alleviate the detrimental effects of pathogens. The present study was conducted to evaluate the effects of different priming methods in the context of resistance to *Aspergillus niger* in wheat (*Triticum aestivum* L.). Here, we show that different priming treatments—viz., hydropriming, osmotic priming, halopriming, and hormonal priming techniques can induce disease resistance by improving the biochemical contents of wheat, including chlorophyll, protein, proline, and sugar. In addition, physiological parameters—such as root length, shoot length, fresh and dry root/shoot ratios, and relative water content were positively affected by these priming methods. In essence, hydropriming and osmotic priming treatments were found to be more potent for enhancing wheat biochemical contents, along with all the physiological parameters, and for reducing disease severity. Hydropriming and osmotic priming significantly decreased disease severity, by 70.59–75.00% and 64.71–88.33%, respectively. RT-PCR and quantitative real-time PCR analyses of potentially important pathogenesis-related (PR)-protein genes (*Thaumatin-like protein* (*TLP*), *chitinase,* and *β-1,3-glucanase*) in primed plants were evaluated: *β-1,3-glucanase* was most highly expressed in all primed plants; *Chitinase* and *TLP* exhibited higher expression in hormonal-, halo-, osmotic-, and hydro-primed plants, respectively. These results suggest that the higher expression of *β-1,3-glucanase, TLP*, and *chitinase* after hydropriming and osmotic priming may increase disease resistance in wheat. Our study demonstrates the greater potential of hydropriming and osmotic priming for alleviating stress caused by *A. niger* inoculation, and enhancing resistance to it, in addition to significantly improving plant growth. Thus, these priming methods could be beneficial for better plant growth and disease resistance in other plants.

## 1. Introduction

Priming is a seed treatment in which seeds are first soaked and then dried to their original weight, during which time germination continues, but radicle protrusion does not occur [1]. Seed priming offers the following advantages: improved, uniform, and fast emergence of seedlings; healthier grain; excessive vigor; and better straw yield, tillering, allometry, and harvest index in floriculture [2], vegetables [3,4] and field crops [5,6,7,8,9]. Various seed treatment techniques have been established, such as osmotic priming, hydropriming, halopriming, thermopriming, and hormonal priming. In hydropriming, pre-germination starts, but actual germination does not occur. Hydro-primed plants tolerate dryness, and the negative effects of pests are decreased by the faster emergence of seedlings [10,11]. Hydropriming enhances seedling growth in rice (*Oriza sativa* L.), corn (*Zea mays* L.), chickpea (*Cicer arietinum*), and mung bean (*Vigna radiata*) seeds [12,13,14]. Hydropriming can be a cheap and easy seed invigoration treatment for wheat, especially in salinity and drought stresses.

In osmotic priming, the seeds are soaked in a low osmotic potential solution, having chemicals like polyethylene glycol (PEG), menthol, chemical fertilizers, sugar, glycerol, and sorbitol [15]. Osmotic priming has been known to improve seed dormancy, and to enhance vigor in soybean (*Glycine max* L.) [16] and tomato (*Solanum lycopersicum*) [17]. PEG solution enhances the emergence percentage and the homogeneity of germination, and increases water absorbance by the seeds, and the development of the shoot and radicle [15,18]. In abiotic stress conditions, cellular stability is maintained by metabolic osmo-regulators, such as glycerol, mannitol, and trehalose, which are well-known osmo-conditioners [19]. Few reports have concluded that osmotic priming agents play a key role in activating crop disease resistance [19]. In wheat, powdery mildew caused by *Blumeria graminis* is controlled by trehalose, which induces systemic acquired resistance [20].

During host–pathogen interactions, pathogenesis-related (PR) proteins are produced: these proteins are encoded by the host plant, but they are induced specifically in pathological or related situations [21]. PR proteins are of paramount importance, as they increase plant resistance to pathogens. Thaumatin-like proteins (TLPs) are important PR-proteins (PR-5), consisting of 200 amino acid residues [22,23,24]. TLPs are produced in plants: they protect the plants from the harmful effects of phytopathogens, stresses, and elicitors, and are also involved in a wide range of developmental signals. The antifungal property of TLPs renders them useful in genetic engineering to produce disease-resistant plants [25,26,27,28]. The role of TLPs in resistance to several basidiomycete fungi—including *Rhizoctonia solani*, *Lentinula edodes* (Berk.), and *Irpex lacteus* (Fr.)—has been reported [29]. Despite exhibiting resistance to biotic stresses, TLPs also confer resistance to abiotic stress conditions [30].

Chitinase is another PR protein (PR-3) expressed in response to a variety of stresses [31]. Chitinase has antifungal activities against plant pathogenic fungi, such as *Fusarium oxysporum*, *Botrytis cinerea*, *Rhizoctonia solani, F. udum*, *Alternaria* sp., *Bipolaris oryzae*, *Curvularia lunata*, and *Mycosphaerella arachidicola* [32,33,34]. The mode of action of PR-3 proteins is relatively simple, e.g., chitinases cleave the chitin polymers of the cell wall in situ, leading to a compromised cell wall that renders fungal cells osmotically sensitive [35]. Another highly complex gene family is the plant β-1,3-glucanase (β-1, 3-G); β-1,3-glucanases play a role in developmental processes and pathogen defense responses [36]. The expression of these genes is triggered by plant hormones, which also affect germination [37]. β-1,3-glucanases are well-recognized PR proteins, which belong to the PR-2 protein family. These PR proteins are strongly induced in response to wounds or infection by viral, bacterial, and fungal pathogens [38,39]. This study aimed to ascertain whether improvement in plant growth and disease resistance could be induced by using different priming techniques. For that purpose, the present study was designed to investigate the role of hydropriming, osmotic priming, halopriming, and hormonal priming in response to *A. niger* inoculation in wheat (*T. aestivum* L.). We found that, of all the priming methods, hydropriming and osmotic priming had the most significant effect on growth and development, decreasing disease severity, and increasing resistance to *A. niger* in wheat: this is most probably due to the higher expression of genes (in hydropriming and osmotic priming) involved in plant defense mechanisms, and their role in disease resistance.

## 2. Materials and Methods

### 2.1. Seed Collection and Preparation

#### 2.1.1. Seed Sterilization

Healthy seeds of the susceptible wheat cultivar “Sahar” were obtained from the National Seed Corporation, Fatteh Jhang, and Rawalpindi, Pakistan. The seeds were surface-sterilized, by being soaked in 70% ethanol for 3 min, washed thoroughly with sterilized distilled water many times, and then dried.

#### 2.1.2. Seed Priming

Four priming methods were used for comparative analysis. In each treatment, 20 g (g) of wheat seeds was used. The osmotic priming technique employed 30 g of polyethylene glycol (PEG 6000), which was dissolved in 100 mL of distilled water. The wheat seeds were soaked in PEG solution for 2–3 days at room temperature, dried to their original weight under shade, and used for sowing [40]. For the hydropriming, the seeds were soaked in distilled water for 24 h at room temperature: these seeds were re-dried to their original weight under a shade with continuously passing air [40]. For the hormonal priming, the wheat seeds were soaked in 200 mL of hormonal solution (100 ppm solution of Indole acetic acid (IAA)) for 12 h at room temperature; then, the seeds were re-dried to their original weight, under shade, and used for sowing. For the halopriming, the seeds were primed in 100 mL of NaCl solution (100 mM) for 12 h, and allowed to air-dry for 12 h at room temperature before sowing.

#### 2.1.3. Seed Sowing and Germination

After priming, the seeds were sown in plastic pots containing sterilized soil, and were kept under controlled conditions in a growth chamber at 20–25 °C day/night temperature, 60% relative humidity, and 14/10 hrs light-and-dark periods. Ten to fifteen seeds were sown in each pot. Non-treated seeds were used as the control.

### 2.2. Fungus Inoculum Preparation

A fresh culture of *A. niger* was obtained from the National Agricultural Research Centre (NARC), Islamabad, and observed under a microscope for confirmation. Using a sterilized spatula, the fungus was transferred to Czpeck media. The flasks were incubated in a shaker incubater (200 rpm) at 30 °C. After 3 days, the number of spores was calculated by hemocytometer, and adjusted to 10^6^ spores/mL concentration. The spore suspension was filtered using a muslin cloth, and the filtrate was used for further foliar and systemic inoculations.

#### Fungus Inoculation

Two methods were used for fungus inoculation. In the foliar (surface) inoculation method, spore suspension (10^6^ spores/mL) was sprayed on 8–10-day-old plants, with the help of a spray bottle. For one week post-inoculation, the symptoms were observed every 24 h. For systemic inoculation, sorghum seeds were used to completely disperse the fungus in the soil. The sorghum (*Sorghum bicolor*) seeds were sterilized in 70% ethanol, washed three times with distilled water, and soaked overnight in distilled water. The seeds were then dried, autoclaved, and soaked in spore suspensions for 5–7 days [41]. The inoculated sorghum seeds were isolated from the spore suspension, re-dried under shade, and 2 g of sorghum seeds was added to 1 kg of soil, which was used to grow the primed wheat seeds. In addition, non-treated sterilized sorghum seeds were used as a negative control.

### 2.3. Disease Severity Analysis

Disease symptoms were evaluated and defined by two different methods. In the first method, total leaf area and infected part were measured, and disease severity was calculated in percentage, using the following formula [42]:

$$\mathrm{Disease}\;\mathrm{severity}=\frac{\mathrm{Area}\;\mathrm{of}\;\mathrm{plant}\;\mathrm{tissue}\;\mathrm{affected}\;\mathrm{by}\;\mathrm{disease}}{\mathrm{Total}\;\mathrm{area}}\times 100$$

In the second method, a visual assessment of wilting was performed after foliar and systemic inoculations, by following standard scaling [43,44,45].

### 2.4. Determination of Biochemical Contents

Different biochemical contents were investigated in the primed plants in response to fungal inoculation. The sugar contents of the leaves were determined by following the method of [46]. The protein, proline, and chlorophyll contents were determined by following the methods of [47,48,49], respectively.

### 2.5. Analysis of Physiological Parameters

Various physiological parameters were measured to evaluate the effectiveness of different priming techniques in response to fungal inoculation: in this respect, the lengths of freshly harvested shoots and roots were measured with measuring tape, and the root/shoot ratio was calculated. The fresh plant samples were kept in an oven at 70 °C for 72 h, in order to analyze the dry root/shoot ratio [50]. The relative water content of the leaves was measured after the different priming methods and induction of biotic stress by the method of [51].

### 2.6. RNA Extraction, Quantification, and cDNA Synthesis

The total RNA from the leaves was extracted by using a ThermoFisher scientific^®^ Gene JET plant RNA purification kit, according to the manufacturer’s protocol. The RNA concentration was calculated by Nanodrop, and was utilized for cDNA synthesis, using a ThermoFisher scientific^®^ cDNA synthesis kit.

### 2.7. Primer Designing and RT-PCR

RT-PCR (BIO-RAD) was performed, to examine the expressions of thaumatin-like protein, β-1,3-glucanase and chitinase genes. Total cDNA was used as a template. The primers used in this experiment are given in Table 1. PCR was carried out in a 25 µL reaction mixture comprising 16 µL of water, 2.5 µL of buffer, 1.5 µL of MgCl_2_, 1.5 µL of dNTPs, 0.5 µL of Taq, 1 µL of template, and 1 µL of both forward and reverse primers. The thermal profile was as follows: 5 min at 94 °C, 25 cycles of 40 s at 94 °C, 1 min at 49 °C, 1 min at 72 °C, and a one-step final extension of 5 min at 72 °C.

### 2.8. Real-Time PCR Analysis

Quantitative real-time PCR was carried out, using the Applied Biosystems 7300 Real-Time PCR System. The PCR was performed using 3 µL of first strand cDNAs and SYBR Green PCR Master Mix (ThermoScientific^®^, Waltham, MA, USA) under the following conditions: initial denaturation at 95 °C for 1 min; 40 cycles of denaturation at 95 °C for 15 s; annealing at 49 °C for 15 s; and extension at 72 °C for 45 s. Data were normalized to the housekeeping Actin gene.

### 2.9. Statistical Analysis

All the experiments were carried out in triplicates (*n* = 3). Microsoft Excel 365 software was used for compiling the experimental data, to form a database for further analysis. All the data were evaluated by one-way ANOVA, and for the graphical illustrations and Tukey’s HSD test to examine the difference among treatment means (*p* ≤ 0.05), the Origin software (Version 2022, OriginLab Corporation, Northamptom, MA, USA) was used.

## 3. Results

### 3.1. Biochemical Content Analysis of Primed Plants in Response to Fungal Stress

#### 3.1.1. Proline

**Figure 1:**
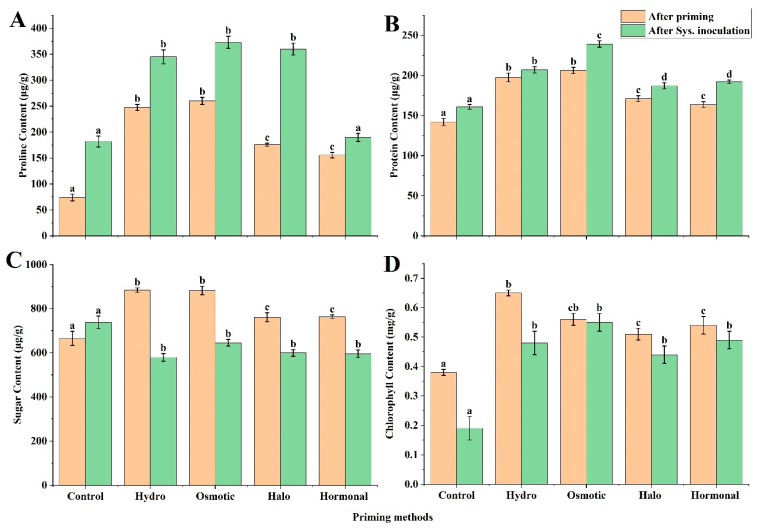
Biochemical contents of wheat under hydropriming, osmotic priming, halopriming, and hormonal priming: (**A**) proline content, (**B**) protein content, (**C**) sugar content, (**D**) chlorophyll content. The mean values with different letter(s) indicate significant differences at *p* ≤ 0.05. Vertical bars represent standard deviation of means (*n* = 3). Sys. inoculation: Systemic inoculation.

The seed priming exhibited a positive effect, by stimulating all the biochemical parameters under both the priming as well as the systemic inoculation conditions. Proline performs its function as a beneficial solute under normal conditions, and as stress tolerance in non-healthy conditions [52]. In our experiment, the total proline content was significantly increased at the seedling stage after each priming treatment, as compared to the control: this significant increase was more pronounced in osmotic and hydro-primed plants—71.56% and 70.09%, respectively—followed by halo-primed and hormonal-primed plants—57.88 and 52.44%, respectively, compared to the control. After systemic inoculation of *A. niger*, the highest increase in proline content was observed in osmotic and halopriming—51.26% and 49.50%, respectively—followed by hydropriming and hormonal priming—47.30% and 4.11%, respectively, compared to the control (Figure 1A).

#### 3.1.2. Protein Content

Production of protein in stress conditions is mainly associated with plant defense responses against fungi [53]. In each priming treatment, protein content was observed to be significantly more increased than in the control. The hydro and osmotic-primed plants had no significant differences in protein content, but when compared to the halo-primed, hormonal-primed, and control plants, a significant change was observed. However, osmotic priming, hydropriming, halopriming, and hormonal priming enhanced protein content by 31.09%, 28.05, 17.00%, and 13.25%, respectively. Moreover, in systemic inoculation of *A. niger* also, a significant increase in protein content was recorded, as compared to the control in all groups. Overall, 32.93% and 22.27% increases in the protein content were observed in osmotic priming and hydropriming, followed by hormonal priming (16.29%) and halopriming (14.00%), compared to the control (Figure 1B).

#### 3.1.3. Sugar Content

Sugar is considered a primary source of energy, which acts as a building block for providing defense-responsive material in plants [54]. Our results showed that the primed plants contained more soluble sugar in their leaves than non-primed and inoculated primed plants. In essence, the osmotic-primed and hydro-primed plants showed a significant increase in sugar content, increasing by 24.60% and 24.75%, respectively, while the halo-primed and hormonal-primed plants exhibited 12.56% and 12.87% increases in sugar contents, respectively, compared to the control plants. By contrast, the systemic inoculation of *A. niger* resulted in a significant drop in sugar content in all primed plants as compared to the control, where 14.32%, 23.07%, 23.92%, and 27.45% greater reductions in sugar content were observed for osmotic-, halo-, hormonal-, and hydro-primed plants, respectively, than in the control (Figure 1C).

#### 3.1.4. Chlorophyll Content

The photosynthetic capacity of plants is determined by their leaf chlorophyll content and measurement [55]. The results of the present study revealed that seed priming exerted a positive effect on the chlorophyll content. The hydro-primed plants showed the highest increase in chlorophyll content, of 41.54%, followed by the osmotic-, hormonal-, and halo-primed plants, which enhanced chlorophyll content by 32.14%, 29.63%, and 25.49%, respectively, as compared to the control. Similarly, under systemic inoculation, all the primed plants showed a significant increase in chlorophyll content, as compared to the control (Figure 1D). However, osmotic priming exhibited the highest chlorophyll contents (65.45%), followed by hormonal priming (51.22%), hydropriming (60.42%), and halopriming (56.82%), compared to the control.

### 3.2. Response of Wheat Physiological Parameters to Different Priming Treatments

#### 3.2.1. Relative Water Content (RWC)

**Figure 2:**
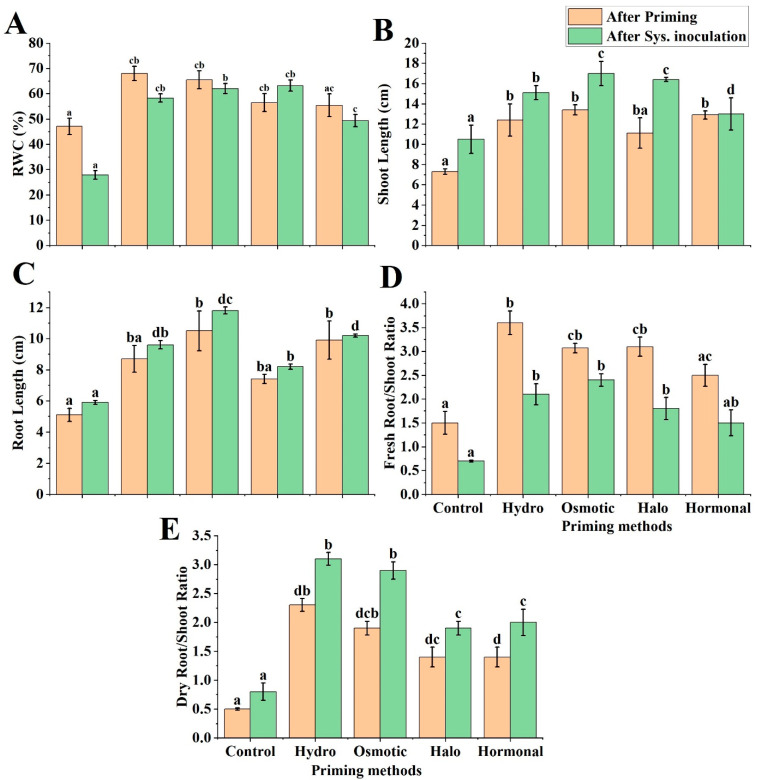
Physiological parameters of wheat under hydropriming, osmotic priming, halopriming, and hormonal priming: (**A**) relative water content (RWC); (**B**) shoot length; (**C**) root length; (**D**) fresh root/shoot ratio; (**E**) dry root/shoot ratio. The mean values with different letter(s) indicate significant differences at *p* ≤ 0.05. Vertical bars represent standard deviation of means (*n* = 3). Sys. inoculation: Systemic inoculation.

In the context of RWC, all the primed plants showed a significant increase in RWC, compared to the non-primed plants; however, the greatest increases in RWC—of 30.74%, 28.09%, 17.08%, and 14.98%, for hydropriming, osmotic priming, halopriming, and hormonal priming, respectively—were observed in comparison to the control. A similar trend of increased RWC was also observed in systemic inoculation of *A. niger* in wheat plants, wherein a significantly greater increase in RWC was observed in all priming treatments than in the control (Figure 2A): the halopriming showed the highest RWC (55.85%), while 55.00%, 52.14%, and 43.52% increases were noted for osmotic priming, hydropriming, and hormonal priming, respectively.

#### 3.2.2. Shoot Length

**Figure 3:**
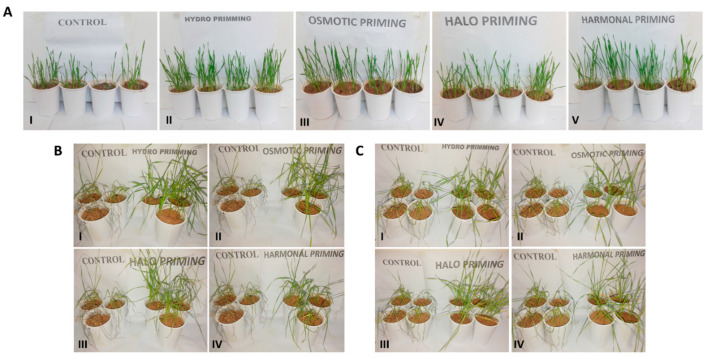
Effects of different priming methods on the growth of wheat plants. (**A**) (**I**): control; (**II**): hydropriming; (**III**): osmotic priming; (**IV**): halopriming, (**V**): hormonal priming. (**B**) Disease severity after foliar inoculation. (**I**): control vs. hydropriming; (**II**): control vs. osmotic priming; (**III**): control vs. halopriming; (**IV**): control vs. hormonal priming. (**C**) Disease severity after systemic inoculation. (**I**): control vs. hydropriming; (**II**): control vs. osmotic priming; (**III**): control vs. halopriming; (**IV**): control vs. hormonal priming.

The application of different priming techniques stimulated shoot growth. An increase in shoot length was significant in plants subjected to all priming treatments, except halopriming, as compared to the control plants (Figure 3A). In principle, the osmotic priming exerted the highest shoot length (45%) compared to the control, while the hormonal priming exhibited a 43.41% increase, the hydropriming a 41.13% increase, and the halo priming a 34.23% increase in shoot length (Figure 2B). Similarly, the shoot length was significantly increased in all primed plants, in comparison to non-primed plants, after systemic inoculation of *A. niger*, where the maximum increases in shoot length—i.e., 38.24%, 35.98%, 30.46, and 19.23%—were recorded for osmotic priming, halopriming, hydropriming, and hormonal priming, respectively.

#### 3.2.3. Root Length

All the priming treatments exhibited a pattern of increase in root length similar to that of shoot length. The highest increases—of 51.43% and 48.48%, respectively—were observed in the root length of plants subjected to osmotic priming and hormonal priming, followed by hydropriming and halopriming, with increases of 41.38% and 31.08%, respectively. Similarly, all primed plants revealed a significant increase in root length, in comparison to the control, after systemic inoculation, where the maximum root length was recorded for osmotic-primed (50.00%) and hormonal-primed plants (42.16%) (Figure 2C).

#### 3.2.4. Fresh and Dry Root/Shoot Ratio

The fresh plant root/shoot ratio was significantly increased in all primed plants, while a non-significant increase was observed in hormonal priming, as compared to the control. With respect to the fresh root/shoot ratio, hydropriming presented the highest increase—of 58.33%—while 51.61%, 51.14%, and 40.00% increases were recorded for halo-, osmotic-, and hormonal-primed plants. The same tendency of increase in the fresh root/shoot ratio was observed after systemic inoculation, where 70.83-enhanced, 66.67%-enhanced, 61.11%-enhanced, and 53.33%-enhanced fresh root/shoot ratios were observed for osmotic priming, halopriming, hydropriming, and hormonal priming (Figure 2D).

In addition, the results exhibited a similar trend of increase in dry root/shoot ratio in all the primed plants: however, this increase was more significant in the hydro-primed plants, whose dry root/shoot ratio increased by 78.26%, while the dry root/shoot ratio of the osmotic-, halo-, and hormonal-primed plants showed 73.68%, 64.29%, and 64.29% increases, respectively, compared to the control plants. Furthermore, in the case of systemic inoculation of *A. niger*, hydropriming and osmotic priming showed the highest increase in dry root/shoot ratio (74.19 and 72.41%, respectively), followed by halopriming and hormonal priming, with enhanced dry root/shoot ratios of 57.89% and 60.00%, respectively, as compared to the control (Figure 2E).

### 3.3. Disease Severity Analysis

#### 3.3.1. Foliar Inoculation

**Figure 4:**
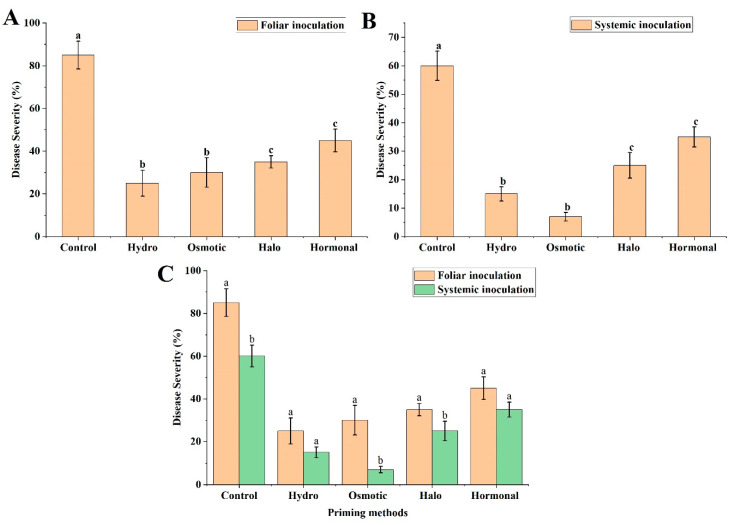
Disease severity analysis of wheat plants in response to hydropriming, osmotic priming, halopriming, and hormonal priming. (**A**) Disease severity analysis after foliar inoculation of *A. niger*. (**B**) Disease severity analysis after systemic inoculation of *A. niger*. (**C**) Disease severity comparison between foliar and systemic inoculation. The mean values with different letter(s) indicate significant differences at *p* ≤ 0.05. Vertical bars represent standard deviation of means (n = 3).

Our results revealed that the foliar inoculation of *A. niger* induced a drastic disease severity in non-primed (control) plants; however, it was observed that the priming treatments significantly reduced disease severity, by alleviating the stress caused by *A. niger* inoculation. Among the priming treatments, hydropriming and osmotic priming showed the maximum decreases in disease severity, of 70.59% and 64.71%, respectively. Halo- and hormonal-primed plants also showed pronounced reductions in disease severity, of 58.82% and 47.06%, respectively, in comparison to the control plants. In general, hydropriming and osmotic priming were observed to be more effective in reducing disease severity, in comparison to halopriming and hormonal priming (Figure 3B and Figure 4A).

#### 3.3.2. Systemic Inoculation

Similarly, the non-primed (control) wheat plants subjected to systemic inoculation showed acute disease severity, with drastically reduced growth. In the case of the primed plants, however, the hydro- and osmotic-primed plants were found to be the most resistant, significantly reducing disease severity by 75.00% and 88.33%, respectively, as compared to the control, while halopriming and hormonal priming showed comparatively less resistance than osmotic priming and hydropriming (Figure 3C and Figure 4B). However, both halopriming and hormonal priming also induced considerable reduction in disease severity, i.e., 58.33% and 41.67%, respectively, compared to non-primed plants.

#### 3.3.3. Comparison of Foliar and Systemic Inoculation

In this study, we obtained promising results with respect to disease severity reduction for the systemic inoculation method, in comparison to the foliar spray method. Both methods were applied for the same length of time, i.e., 2 weeks, and disease symptoms appeared more rapidly in the foliar spray method than in the systemic method. The results revealed that the plants treated with systemic fungus inoculation exhibited more resistance to disease in comparison to the foliar spray technique. In particular, osmotic priming and halopriming in systemic inoculation presented significant differences in reducing disease severity—by 76.67% and 40.00%, respectively—compared to foliar-sprayed plants of the same group. In addition, halopriming and hormonal priming also revealed a considerable decrease in disease severity reduction—of 28.57% and 22.22%, respectively—when compared to foliar-sprayed plants of the same treatment (Figure 4C).

#### 3.3.4. Visual Assessment of Wilting

**Figure 5:**
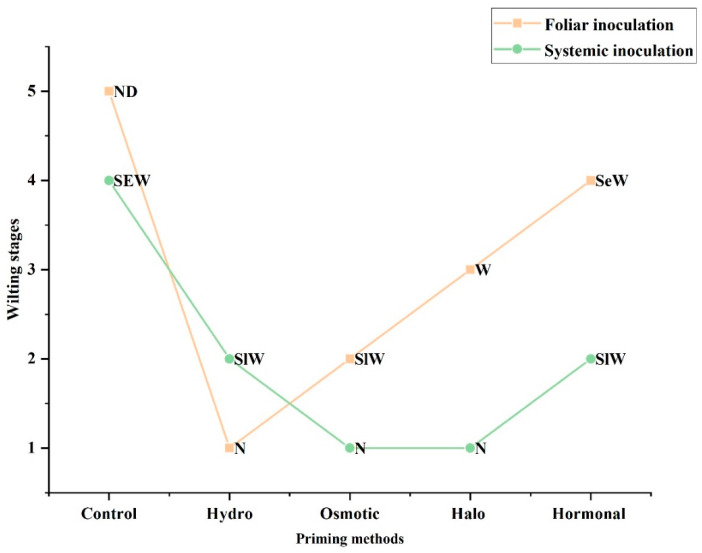
Measurement of disease severity after foliar and systemic inoculation, by visual assessment of wilting. Different wilting conditions are described as normal (N), slightly wilted (SlW), wilted (W), severely wilted (SeW), nearly dead (ND), and dead (D).

Visual assessment of wilting also revealed the same pattern as described above for the disease severity percentage. After foliar inoculation of *A. niger*, the control plants were found to be nearly dead, while the hydro- and osmotic-primed plants were normal, but slightly wilted. The halo-primed plants showed wilting (W), while the hormonal-primed plants were wilted severely (Figure 3B and Figure 5). Likewise, the same pattern of visual assessment of wilting was observed with systemic inoculation, where the control plants were found to be severely wilted, while the hydro- and hormonal-primed plants were wilted slightly; however, the osmotic- and halo-primed plants seemed to be normal (Figure 3C and Figure 5).

### 3.4. Expression Profiling of TLP, Chitinase, and β-1,3-glucanase Genes

**Figure 6:**
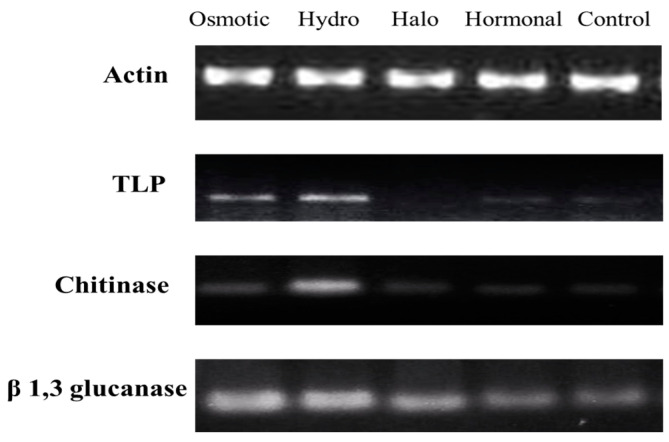
Expression profiling of *TLP, Chitinase, and β-1, 3-glucanase* by RT-PCR.

**Figure 7:**
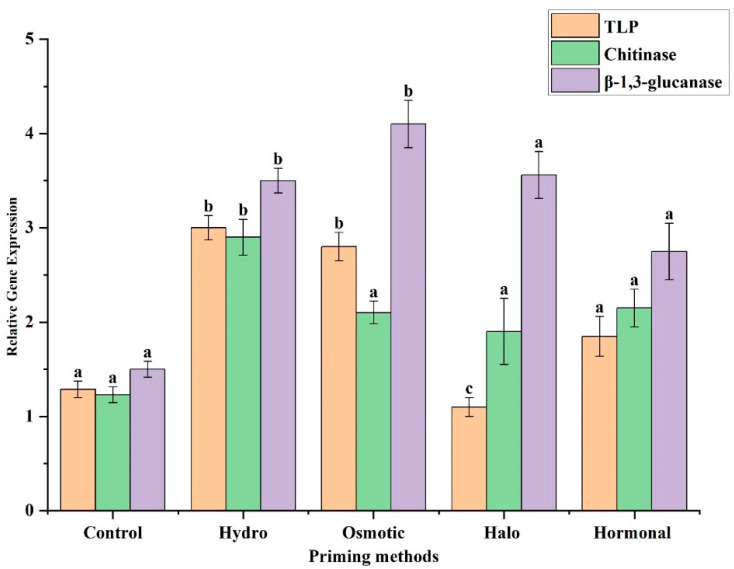
Relative expression of *TLP, Chitinase*, and *β-1,3-glucanase*, obtained through quantitative real-time PCR analysis. The mean values with different letter(s) indicate significant differences at *p* ≤ 0.05. Vertical bars represent standard deviation of means (*n* = 3).

TLP gene expression was down-regulated in halo-primed plants compared to the control, while osmotic- and hydro-primed plants showed significantly higher expression of *TLP*. In halo-primed plants, almost no detectable expression of the *TLP* gene was seen. The expression profile of *TLP* in RT-PCR and qRT-PCR was comparable (Figure 6 and Figure 7). Both RT-PCR and qPCR showed that *chitinase* gene expression was significantly increased in hydropriming compared to the plants treated with osmotic priming, halo priming, and hormonal priming (Figure 6 and Figure 7). RT-PCR and qPCR results also confirmed that *β-1,3-glucanase* was highly expressed in hydro- and osmotic-primed plants compared to non-primed plants, while halo- and hormonal-primed plants also showed a considerably increased expression of *β-1,3-glucanase*; however, the change was not as significant as compared to the control (Figure 6 and Figure 7). Overall, the analysis of the relative gene expression indicated that *β-1,3-glucanase* presented a significant role in inducing resistance to *A. niger* under each priming treatment, followed by *chitinase* and *TLP*, which played a considerable role in resistance to *A. niger* under halopriming and hormonal priming, and under hydropriming and osmotic priming, respectively.

## 4. Discussion

Seed priming has been extensively used for the improvement of seed quality yield, and to lower seedling protrusion time. Different priming techniques are being used in this regard, all of which have their own advantages [56]. This study was conducted to evaluate the potential of different priming techniques—i.e., hydropriming, osmotic priming, halopriming, and hormonal priming—to not only contribute to gain in seed growth and health, but also confer resistance against a pathogenic fungus, *A. niger*. To evaluate disease severity and resistance, we conducted disease severity analysis, as described above, and measured the expression level of the genes—namely *chitinase, TLP*, and *β-1,3-glucanase*—which mainly contribute to the host resistance to pathogens.

We evaluated biochemical and physiological parameters after treatment with different priming techniques. In the present study, higher proline content was observed in all priming treatments, but this effect was more pronounced in hydropriming and osmotic priming, which enhanced proline content by 70.09% and 71.56% more than non-primed plants (control) (Figure 1A). It has been shown that under various stress conditions—e.g., high salinity, drought, and biotic stress—proline accumulates in high concentration [57,58,59]. Previous studies on coriander (*Coriandrum sativum*) [60] and sorghum [61] have also described the increased synthesis of proline due to priming. In the case of systemic inoculation, the hydro-, osmotic-, and halo-primed plants showed, by increased proline content, better disease resistance to fungus inoculation (47.30%, 51.26%, and 49.50%, respectively) (Figure 4). Similarly, a significant increase in proline was noted in *Brassica napus* during osmotic priming [62]. Manghwar et al. [28] also observed enhanced proline content in wheat under *Fusarium equiseti* stress. Proline is a compatible solute, usually accumulated under stress in plants, and acts in osmotic adjustment [57,63]. The results of the present study showed significantly increased protein content with all priming treatments compared to the control. Comparatively, all the primed plants inoculated with *A. niger* resulted in higher protein production than non-inoculated primed plants. The findings of [64] also showed the positive effect of priming on the protein contents of the common bean: fungal inoculation led to an overall increase in protein content and a decrease in sugar contents, which is a sign of the stimulation of osmotic material synthesis under stress conditions [65].

Moreover, an increase in sugar content after priming may be because leaves synthesize more soluble sugars after seed priming. The same beneficial effect was found in safflower (*Carthamus tinctorius*) [66], wheat [67], pepper (*Capsicum annuum* L. var Chargui) [68], and barley (*Hordeum vulgare* L.) [69]: this increase may be due to increased α-amylase activity [70]. Sugar content in our study was slightly decreased in response to fungal stress in all the pre-treated plants. Other studies have also confirmed the decrease in sugar content of primed plants after stress conditions [71]. Generally, some pathogenic infections bring changes to the photosynthetic rate and respiratory pathway, and cause fluctuation in sugar content [72,73,74]. The priming treatments in our study also led to increased chlorophyll content (Figure 1D). A significant increase in chlorophyll contents has been observed after osmotic priming and hydropriming. The study reported 43% and 100% increases in chlorophyll a and b contents, respectively, after priming [75]. Another study, of water, auxin, and gibberellins priming, has been reported to uplift chlorophyll content in soybean [76]. Related results after different priming methods have been observed in rice [77] and coriander [78]. An increase in the chlorophyll content of inoculated primed plants indicates the possible role of priming in disease resistance. The decrease in chlorophyll content of non-treated control plants after systemic inoculation of *A. niger* suggests the positive role of seed priming in maintaining chlorophyll content and disease resistance.

In the present study, higher RWCs were observed after seed treatments. Of all the treatments, the hydro- and osmotic-primed plants showed the highest accumulation of RWCs (Figure 2A). The same results were reported by Namdari and Baghbani [79] and by Mahboob et al. [80], who reported higher water content in *Vicia dasycarpa* and *Zea mays* with hydropriming and osmotic priming, respectively. Our findings revealed an increase in shoot and root length after priming compared to the control, which is supported by the findings of Dessalew et al. [4] and Kumar and Rajalekshmi [81]. Anwar et al. [62] observed an increase in root length in primed seeds in comparison to their control, and suggested that it could be because of embryo cell wall extensibility. In addition, it has been reported that after priming, cell division increases in the apical meristem in roots, leading to an increase in plant growth [82].

The present study showed the beneficial effects of hydropriming and osmotic priming on shoot length, root length, and fresh and dry root/shoot ratios, in response to fungal attack (Figure 2B–E). The hydro- and halo-primed China aster (*Callistephus chinensis*) plants showed significantly enhanced seed germination percentage, seedling survival, and root/shoot ratio [83]. Bourioug et al. [75] reported that hydropriming and osmotic priming in sunflower (*Helianthus annuus*) promoted overall plant growth and increased grain number and grain yield per plant by 2.5-fold and 3.3-fold, respectively. It has been suggested that seed priming enhances plant growth by decreasing the effect of oxidative reactions triggered by reactive oxygen species (ROS) in plant cells [84,85]. According to Al-Abdalall [86], laboratory treatment of both wheat and barley crops by fungi reduces root and shoot lengths and yield significantly. We also observed a decrease in all these parameters in the control (non-primed) plants after *A. niger* inoculation, in comparison to the primed plants, which could be a reason for providing resistance to the pathogen.

Zida et al. [87] reported that seed priming of sorghum plants exhibited significant increase in crop yield, of 19.6% to 51.7%. In addition, the study described threefold to fivefold decreases in the fungal species, *Curvularia* and *Epicoccum*, respectively. Similarly, Rashid et al. [88] demonstrated that, due to hydropriming, mung bean appeared to be more disease-resistant, by having fewer disease symptoms after being infected with Mung bean Yellow Mosaic Virus (MYMV). Rashid et al. [89] also reported an increase in biomass and grain weight due to priming. Likewise, our results also represent that primed plants have a considerable decrease in disease severity, by having improved biochemical (proline, protein, sugar, and chlorophyll contents) and physiological parameters (fresh root, shoot length, dry root/shoot ratio, and RWC). Foliar inoculation of *A. niger* showed a higher percentage of disease or leaf necrosis in the control (>80% leaf area) (Figure 4). The plants were found to be nearly dead, by visual assessment of wilting, as shown in Figure 5. At the same time, a considerable decrease was observed in disease severity, especially in hydro- and osmotic-primed plants—70.59% and 64.71%, respectively—compared to the control, which could be effective in increasing the yield of the wheat crop. Systemic inoculation also had the same pattern of disease severity, but the capacity of disease accumulation was much less (about 60% in the control, Figure 4B,C) as compared to foliar inoculation, which gives an indication that systemic inoculation might be a vigorous method of pathogen inoculation, to show a more robust response.

Results from RT-PCR and qPCR suggest a possible role of *TLP, chitinase*, and *β-1, 3- glucanase* genes in inducing disease resistance in hydro- and osmotic-primed plants (Figure 7). Higher expression of these genes may increase resistance to *A. niger*. The higher expression of *TLP* genes in plants has been shown to provide enhanced tolerance to fungal pathogens [90,91]. Constitutive expression of *TLP*s is typically absent in healthy plants, but is induced exclusively in response to wounding or pathogenic attack [23,26]. After infecting potato plants with *Phytophthora infestan*s, the *TLP* gene was observed to be up-regulated [92]. We also recorded a significant up-regulation of the *TLP* gene in both hydro- and osmotic-primed plants—suggesting its positive role in disease resistance. *Chitinase* has been reported to have a prominent role in plant defense against fungi [27,28]: this gene is thought to play a dual role in fungal growth inhibition, both by cell wall digestion and by releasing pathogen-borne elicitors that induce further defense reactions in the host [93]. Plants subjected to hydropriming also have higher expression of chitinase, which could possibly be considered highly resistant to disease. It has been shown that due to pathogenic attack, the activity and expression of chitinase are elevated [94]. The best-known examples of protection conferred by transgenic expression of plant antifungal genes are represented by overexpression of *chitinases* and *β-1,3-glucanase*s [28,95]. Importantly, gene expression analysis of the current study revealed that *β-1,3-glucanases* showed the highest expression in each priming treatment, as compared to *TLP* and chitinase: their highest expression was observed in osmotic-primed plants, which resulted in the greatest disease resistance with the lowest disease severity in inoculated wheat plants. These results indicate the possible involvement of β-1,3-glucanase in disease resistance, by inducing its high expression in both hydro- and osmotic-primed plants. In our previous study, we also observed higher expression of β-1,3-glucanase, TLP, and chitinase2, which increased the resistance of wheat plants to *F. equiseti* [28].

## 5. Conclusions

The present study observed the roles of different priming methods—including hydropriming, osmotic priming, halopriming, and hormonal priming—in enhancing resistance to *A. niger* in wheat. All the priming methods used in our study exerted positive effects on plant growth and development, and on resistance to *A. niger*: however, hydropriming and osmotic priming proved to be the best, by significantly improving biochemical (proline, protein, sugar, and chlorophyll contents) and physiological parameters (RWC, root length, shoot length, and fresh and dry root/shoot ratio). In addition, we observed that hydropriming and osmotic priming induced the highest expression of different stress-related genes, such as *TLP, chitinase*, and *β-1, 3-glucanase*: this may be why wheat plants under hydropriming and osmotic priming exhibited the least disease severity, and higher resistance to *A. niger*. Thus, we conclude that hydropriming and osmotic priming may play an important role in reducing the severity of, and resistance to, disease in plants, which could eventually lead to improved crop yield.

## Figures and Tables

**Table 1:** Primers used in this experiment.

S. No.	Protein	Primers
1	Thaumatin-like protein (TLP)	Forward 5′ GCAGTCAAGGCAGTTGGTGGTA 3′, Reverse 5′ GCAGTCAAGGCAGTTGGTGGTA 3′
2	Chitinase	Forward 5′ CGCAGTCACCTAAACCTTCG 3′ Reverse 5′ GCAGTAGCGCTTGTAGAACC 3′
3	β 1,3-glucanase	Forward 5′ CTACAGGTCCAAGGGCATCA 3′ Reverse 5′ CCGGACATTGTTCTGAACCC 3′
4	Actin	Forward 5′ CAAAGAGATCACGGCCCTTG 3′ Reverse 5′ ACTTCATGTGGACAATGCCG 3′

## Data Availability

Not applicable.
